# Anti-VEGF Signalling Mechanism in HUVECs by Melatonin, Serotonin, Hydroxytyrosol and Other Bioactive Compounds

**DOI:** 10.3390/nu11102421

**Published:** 2019-10-11

**Authors:** Ana B. Cerezo, María Labrador, Andrés Gutiérrez, Ruth Hornedo-Ortega, Ana M. Troncoso, M. Carmen Garcia-Parrilla

**Affiliations:** 1 Departamento de Nutrición y Bromatología, Toxicología y Medicina Legal, Facultad de Farmacia, Universidad de Sevilla. C/Profesor García González 2, 41012 Sevilla, Spain; 2 MIB, Unité de Recherche Œnologie, EA4577, USC 1366 INRA, ISVV, Université de Bordeaux, 33822 Villenave d’Ornon, Cedex, France

**Keywords:** VEGF, angiogenesis, melatonin, serotonin, 3-indoleacetic acid, 5-hydroxytryptophol, hydroxytyrosol, PLCγ1, Akt, eNOS

## Abstract

Angiogenesis drives evolution and destabilisation of atherosclerotic plaques and the growth and expansion of tumour cells. Vascular endothelial growth factor (VEGF) is the main endogenous pro-angiogenic factor in humans. The aim was to provide insight into the anti-VEGF activity of bioactive compounds derived from aromatic amino acids (serotonin, melatonin, 3-indoleacetic acid, 5-hydroxytryptophol and hydroxytyrosol). Experiments involved endothelial cell migration (wound-healing assay), the molecular mechanisms (ELISA assay) and the downstream effects (phospholipase C gamma 1 (PLCγ1), protein kinase B (Akt) and endothelial nitric oxide synthase (eNOS) by Western blot) on human umbilical vein endothelial cells (HUVECs). The data suggest for the first time that hydroxytyrosol interacts with surface components of the endothelial cell membrane (, preventing VEGF from activating its receptor. Serotonin and 5-hydroxytryptophol significantly inhibited HUVEC migration (98% and 50%, respectively) following the same mechanism. Conversely to other bioactive compounds, the anti-angiogenic effect of melatonin, serotonin, 3-indoleacetic acid and 5-hydroxytryptophol is not mediated via PLCγ1. However, hydroxytyrosol inhibits PLCγ1 phosphorylation. Additionally, melatonin and serotonin maintained eNOS phosphorylation and hydroxytyrosol significantly activated eNOS—all via Akt. These data provide new evidence supporting the interest in melatonin, serotonin, 3-indoleacetic acid, 5-hydroxytryptophol and hydroxytyrosol for their further exploitation as anti-VEGF ingredients in food.

## 1. Introduction

Worldwide, cardiovascular diseases and cancer are the two main causes of death (31% and 16% of deaths in 2015, respectively) [1,2]. Both diseases share the critical component of forming new blood vessels (angiogenesis), driving both the growth and advance of tumour cells and the evolution and destabilisation of atherosclerotic plaques [3,4]. Moreover, vascular endothelial growth factor (VEGF), which is the main endogenous pro-angiogenic factor in humans [5,6,7,8], has been demonstrated to promote atherosclerotic plaque progression [3,9] and tumoral angiogenesis [10]. Vascular endothelial growth factor works by stimulating its principal receptor VEGFR-2, the latter being crucial for promoting endothelial cell proliferation, differentiation and migration [5,11]. Since VEGF is the main pro-angiogenic factor, VEGF signalling inhibition is a plausible molecular mechanism which demonstrates a direct cause–effect relationship in reducing the risk of cardiovascular disease and cancer.

Proof of the crucial function of VEGF in angiogenesis is the fact that certain drugs currently used to treat cancer, such as bevacizumab—the most widely used at present in treating breast, lung, kidney, liver and colon cancer—consist of anti-VEGF molecule treatment [12,13]. Nevertheless, their long-term use has been associated with serious adverse effects such as hypertension [14,15] due to the fact that VEGF inhibition also drives the inhibition of intracellular proteins such as endothelial nitric oxide synthase (eNOS), responsible for generating nitric oxide which is involved in vasodilation.

Certain bioactive compounds present in the Mediterranean diet, such as polyphenols and indolic compounds, have been demonstrated to inhibit VEGF signalling [16,17,18,19,20]. Hence, polyphenols such as epigallocatechin gallate (EGCG), procyanidin tetramers (dp4), quercetin, etc., present in apples, green tea, onions, or cocoa, have been demonstrated to interact directly with the VEGF molecule, rendering it completely inactive [16,17]. Likewise, Moyle et al. [16] proved that not only did EGCG and dp4 procyanidins inhibit VEGF but they also simultaneously increased eNOS phosphorylation in human umbilical vein endothelial cells (HUVECs), something which would, presumably, increase vasodilation. Our research group has recently shown that certain stilbene compounds present in grapes and wine, such as astringin, pallidol, ω-viniferin and ε-viniferin, also have a potential anti-VEGF effect, with only ε-viniferin and pallidol allowing eNOS phosphorylation [20]. Therefore, these data support that not all bioactive compounds which inhibit VEGF have the ability to simultaneously stimulate eNOS. Hydroxytyrosol, present in olive oil and wine [21,22], has also been shown to potentially inhibit VEGF signalling and HUVEC migration [18]. However, its molecular mechanism and effect on eNOS modulation have not been explored, which is an important factor, as it is a European Food Safety Authority (EFSA) requirement for supporting food bioactive health claims.

Our research group has also recently demonstrated that melatonin, present in an extensive array of foods, such as vegetables and their fruits and seeds, medicinal herbs, and fermented products (tomatoes, pistachio, strawberry, cherry, bread, olive oil, wine, beer, etc.), as well as other bioactive indolic compounds (e.g., serotonin, 3-indolacetic acid), also present in fermented food (wine and beer) [23,24,25,26,27,28,29,30], and one of the serotonin human metabolites (5-hydroxytryptophol) [31,32] possess potential anti-VEGF signalling effects [19]. This research has demonstrated that these indolic compounds present a different anti-VEGF molecular signalling mechanism to that of phenolic compounds; serotonin, 3-indolacetic acid, melatonin and 5-hydroxytryptophol appear to interact with the cell surface components of the endothelial membrane, such as VEGFR-2, among others, preventing VEGF from activating the receptor. Therefore, these data reveal that not all bioactive compounds possess the same anti-VEGF molecular signalling mechanism of action. This work has also proved that melatonin and 3-indolacetic acid inhibit HUVEC migration, demonstrating its anti-angiogenic effect. However, the effect of serotonin and 5-hydroxytryptophol on inhibiting HUVEC migration, and the influence of 3-indolacetic acid, melatonin, 5-hydroxytryptophol and serotonin on modulating the intracellular pathways are both still unexplored.

The aim of the present study was to: (i) provide insight into hydroxytyrosol’s possible anti-angiogenic mechanism of action, (ii) evaluate the anti-angiogenic effect of serotonin and 5-hydroxytryptophol on HUVECs, and (iii) determine the downstream effects (phospholipase C gamma 1 (PLCγ1), protein kinase B (Akt) and eNOS) of bioactive compounds (serotonin, 5-hydroxytryptophol, 3-indolacetic acid, melatonin and hydroxytyrosol) which induce VEGF signalling inhibition. Since these bioactive compounds are present concurrently in the diet, this work aims to evaluate their cocktail effect upon the inhibition of VEGF-induced VEGFR-2 activation.

## 2. Materials and Methods

### 2.1. Cell Culture

The HUVECs (Lonza, Slough, UK) between passages 4 and 5 were kept in endothelial growth medium-2 (EGM-2) (Lonza, Slough, UK) at 37 °C and 5% CO_2_.

### 2.2. Compounds Under Study

Serotonin, melatonin and 3-indolacetic acid were obtained from Sigma–Aldrich (St. Louis, MO, USA). 5-Hydroxytryptophol and hydroxytyrosol were acquired from Cayman Chemical (Ann Arbor, MI, USA).

### 2.3. Treatment of HUVECs

The HUVECs were grown to confluence and exposed to endothelial basal medium (EBM) containing either the individual compounds under study (indolic compounds at 1 mM and hydroxytyrosol at 1 and 50 µM; final concentration of ≤0.1% dimethyl sulfoxide (DMSO)) or DMSO for vehicle controls (≤0.1% DMSO). These compound concentrations were according to the previous anti-VEGF effect reported in the literature [18,19]. The cells were then incubated for 4 h at 37 °C. Stimulation with VEGF (25 ng/mL) (VEGF_165_, R&D Systems, Minneapolis, MN, USA) was then conducted for 5, 10 or 60 min depending on whether VEGFR-2, PLCγ1 or Akt and eNOS phosphorylation, respectively, was being determined. Each sample was analysed in duplicate, and experiments were performed twice. After the treatments, cells were lysed by radioimmunoprecipitation and total protein content quantified by bicinchoninic acid (BCA) assay.

In the case of hydroxytyrosol, three different experimental conditions (previously designed in Moyle et al. [16]) were tested in order to elucidate the molecular mechanism for inhibiting VEGF-induced VEGFR-2 phosphorylation: (1) a mixture of hydroxytyrosol and VEGF 5 min prior to cell treatment with the mixture for other 5 min; (2) cell pre-incubation with hydroxytyrosol for 4 h prior to cell incubation with VEGF for 5 min; and (3) cell pre-treatment with hydroxytyrosol for 4 h, washing passage and stimulation with VEGF for 5 min. Finally, in order to determine the hydroxytyrosol IC_50_ value, experiments were carried out testing six different concentrations (1–100 µM) after 4 h of pre-incubation (experimental design 2).

### 2.4. VEGFR-2 Phosphorylation (ELISA Assay)

The PathScan Phospho-VEGFR-2 (Tyr 1175) sandwich ELISA kit (Cell Signaling Technology, Hitchin, UK) was used to quantify VEGFR-2 activation in protein lysates, the procedure being performed following the manufacturer’s instructions.

### 2.5. Determination of PLCγ1, Akt and eNOS Modulation by Western Blot

Denaturalised protein lysates (25 µg) (with lithium dodecyl sulphate and DTT at 70 °C for 10 min) (Invitrogen, Loughborough, UK) were loaded on NuPAGE 4%–12% Bis-Tris (Invitrogen, Loughborough, UK) gels and separated by electrophoresis. Subsequently, proteins were transferred to 0.2 μm nitrocellulose membranes (Bio-Rad, Hercules, CA, USA) and incubated with anti-phospho antibodies and total proteins of PLCγ1 (Tyr 783; Ref 2821 and 5690), Akt (Ser 473; Ref 4060 and 9272) and eNOS (Ser 1177; Ref 9570 and 9572) (Cell Signaling Technology). The membranes were visualised using secondary anti-rabbit antibodies IgG-HRP (Ref 7074) and SuperSignal West Pico chemiluminescent substrate (Thermo Scientific, Hitchin, UK) on an Amersham Imager 600 station (GE Healthcare live sciences, Marlborough, MA, USA).

### 2.6. Evaluation of Migration (Wound-Healing Assay)

The cell migration assay was performed following the protocol previously described in Cerezo et al. [19]. The HUVECs were placed onto 50 mm imaging dishes (Ibidi, Martinsried, Germany). Once cells reached full confluence, the monolayer was scratched across the centre using a sterile 200 μL pipette tip. Subsequently, the cells were then rinsed twice with PBS to eliminate any dead cells. Serum-free medium containing 1 mM serotonin, 1 mM 5-hydroxytryptophol or vehicle controls were added (final concentration ≤ 0.1% DMSO) for 4 h prior to VEGF stimulation (25 ng/mL) over 24 h. A phase contrast microscope (Nikon, Tokyo, Japan) was used to photograph the wound-gaps. Nis-Elements BR v.4.30.02 software (Nikon, Tokyo, Japan) was utilised to measure the initial and final sizes of each wound. The following formula was used to quantify the migration distance: initial wound size minus final wound size divided by two. Two independent experiments were performed.

### 2.7. Statistical Analysis

Significant differences among samples were analysed using Student’s *t*-test in GraphPad Prism software version 6.01 (GraphPad Software, Inc., San Diego, CA, USA).

## 3. Results

### 3.1. Hydroxytyrosol in the Inhibition of Angiogenesis: Molecular Mechanism

**Figure 1:**
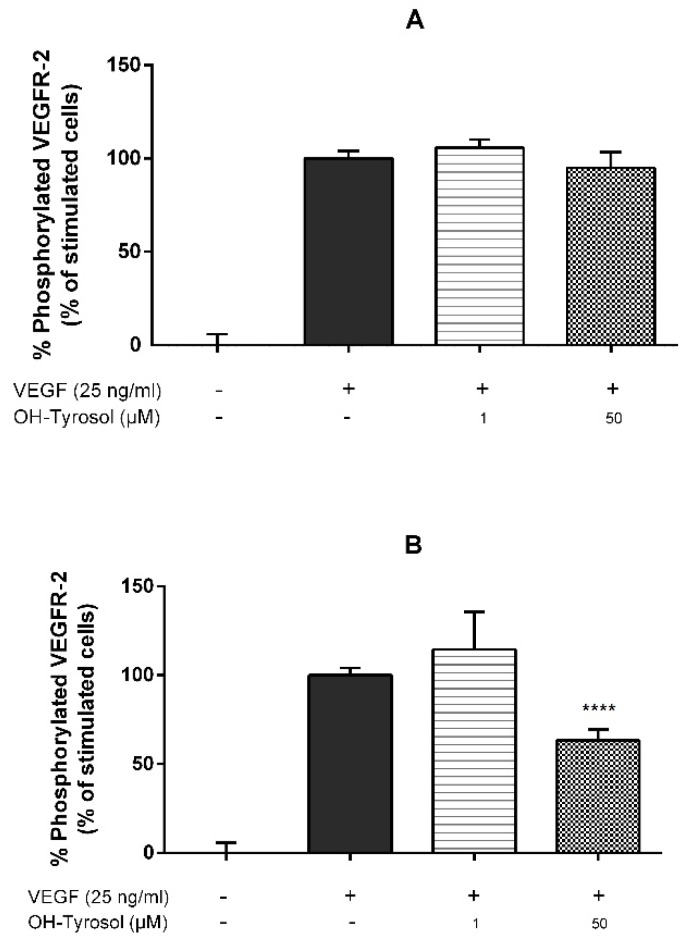

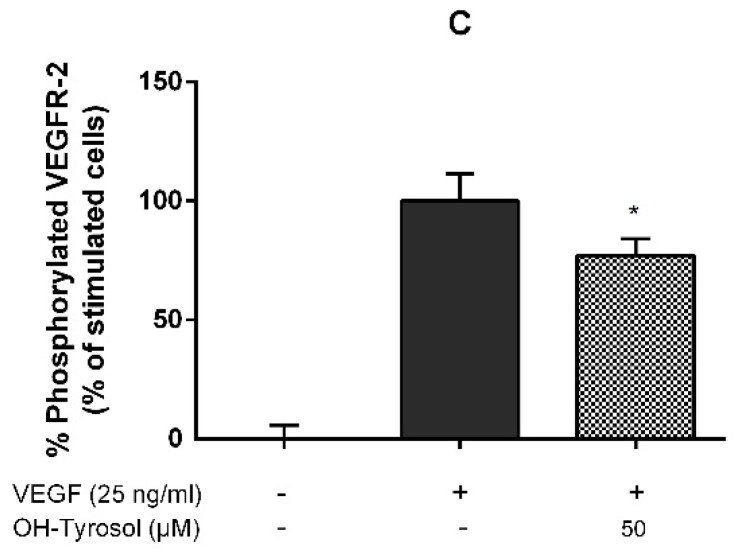
Hydroxytyrosol inhibits vascular endothelial growth factor (VEGF)-induced VEGFR-2 phosphorylation by interacting with cell membrane components. Human umbilical vein endothelial cells (HUVECs) were incubated with 50 μM or 1 μM hydroxytyrosol and VEGF (25 ng/mL). (**A**) Hydroxytyrosol and VEGF were mixed 5 min prior to cells treatment for another 5 min; (**B**) hydroxytyrosol was pre-incubated for 4 h with HUVECs and subsequently incubated with VEGF for 5 min; (**C**) hydroxytyrosol was pre-incubated for 4 h with HUVECs and, after removal of the polyphenol (with PBS), was then stimulated with VEGF for 5 min. p-VEGFR-2 (Tyr1175) was quantified by ELISA essay; * *p* < 0.1, **** *p* < 0.0001 in comparison to the stimulated cells with VEGF alone. Data are expressed as the mean ± SD (*n* = 4). OH-Tyrosol: hydroxytyrosol.

To elucidate hydroxytyrosol’s molecular mechanism, three different experiments were performed (Figure 1). First, either vehicle control or hydroxytyrosol (1 μM and 50 μM) with VEGF (25 ng/mL) were mixed for 5 min prior to treating HUVECs for 5 min. The results in this paper show that the hydroxytyrosol treatment failed to inhibit VEGF-induced VEGFR-2 phosphorylation (Figure 1A). Thus, these results rejected the hypothesis that, like other phenolic compounds such as epigallocatechin gallate or quercetin, among others [16], hydroxytyrosol could interact directly with the VEGF protein.

Secondly, HUVECs were incubated with either vehicle control (≤0.1% DMSO) or hydroxytyrosol (1 μM and 50 μM) for 4 h before stimulating them with VEGF (25 ng/mL) for 5 min. Hydroxytyrosol at 50 μM significantly inhibited VEGFR-2 phosphorylation by 37% compared to control with VEGF only (Figure 1B). These results support the notion that hydroxytyrosol could bind to VEGFR-2 or any of its co-receptors such as heparan sulphate proteoglycans (HSPGs) or neuropilins (NRPs), affecting the activation of VEGFR-2.

Thirdly, HUVECs were incubated with 50 μM hydroxytyrosol (active concentration) for 4 h. The cells were then washed twice with PBS and stimulated with new media containing VEGF only (25 ng/mL) for 5 min. The results proved that hydroxytyrosol still significantly inhibited VEGFR-2 activation by 23% (Figure 1C). The second and third experimental designs led us to hypotheses that hydroxytyrosol could interact both with VEGFR-2 or any of its co-receptors at the extracellular domain. This is probably due to a non-strong binding, which can be removed in the rinsing step, and, in addition, can interact with any sub-cellular kinase, which could explain the remaining 23% of pVEGFR-2 inhibition.

Figure 1B shows that hydroxytyrosol inhibition of VEGF-induced VEGFR-2 phosphorylation appeared to occur in a concentration-dependent manner, which agrees with Lamy et al. [18]. Therefore, the half maximal inhibition (IC_50_) of hydroxytyrosol under the abovementioned 4 h pre-incubation conditions was then determined, giving the result that IC_50_ = 72.4 μM (Table 1).

### 3.2. Anti-Angiogenic Effect of Serotonin and 5-Hydroxytryptophol: Inhibition of HUVEC Cell Migration

**Figure 2:**
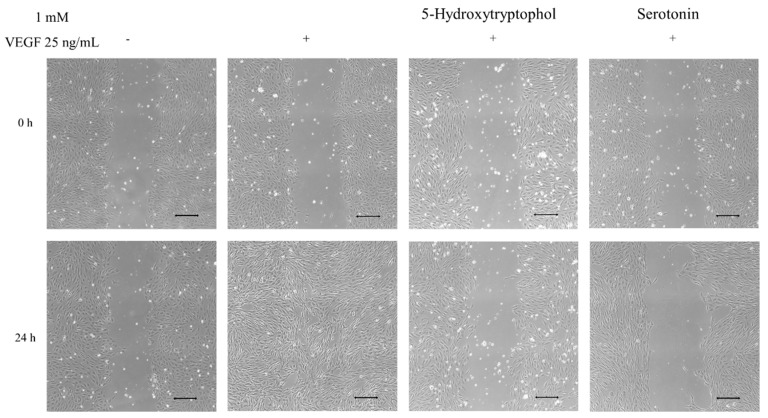
Serotonin and 5-hydroxytryptophol inhibited HUVEC migration. HUVECs were placed onto 50 mm imaging dishes and wounded. Then, they were incubated with 1 mM of 5-hydroxytryptophol and serotonin for 4 h and subsequently stimulated with VEGF. Representative photomicrographs were taken at 0 h and 24 h of VEGF stimulation. Scale bars: 200 μm.

Since serotonin and 5-hydroxytryptophol have been demonstrated to inhibit VEGFR-2 phosphorylation in previous studies [19], their anti-angiogenic effect on HUVEC migration was evaluated by performing wound healing essays. Endothelial cells that were stimulated with VEGF for 24 h refilled the wound closure (Figure 2), whereas cells pre-treated with either serotonin or 5-hydroxytryptophol reduced cell migration by 97% and 50%, respectively, after 24 h (Figure 2).

### 3.3. Downstream Effects of Melatonin, Serotonin, 3-Indolacetic Acid, 5-Hydroxytryptophol and Hydroxytyrosol on PLCγ1, Akt and eNOS Activation

**Figure 3:**
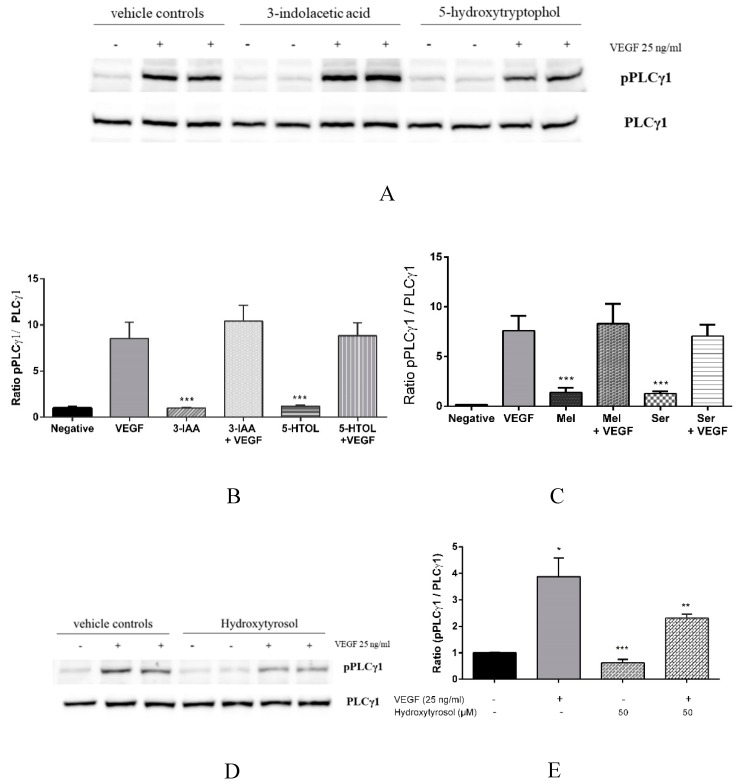
Hydroxytyrosol inhibits PLCγ-1 phosphorylation. HUVECs were treated with (**A**–**C**) 3-indolacetic acid (1 mM), 5-hydroxytryptophol (1 mM), melatonin (1 mM) and serotonin (1 mM), and (**D** and **E**) hydroxytyrosol (50 μM) for 4 h and then incubated with VEGF (25 ng/mL) for 10 min. Western blot membranes were incubated with anti-PLCγ-1 and anti-p-PLCγ-1 antibodies. Data representations of the p-PLCγ-1/PLCγ-1 ratio are expressed as the mean ± SD (*n* = 4). * *p* <0.05; ** *p* < 0.01; *** *p* < 0.001 versus VEGF alone. IAA: 3-indolacetic acid; 5-HTOL: 5-hydroxytryptophol; Mel: melatonin; Ser: serotonin.

Vascular endothelial growth factor is known to mediate different events in angiogenesis through a complex intracellular signalling cascade [33]. Since 3-indolacetic acid, melatonin, 5-hydroxytryptophol, serotonin and hydroxytyrosol have been demonstrated to inhibit VEGF-induced VEGFR-2 phosphorylation [19] (Figure 1) and cell migration [18,19] (Figure 2), determining which downstream substrates could be implicated in the inhibitory activity of the compounds under study is of great interest. First, we evaluated whether the anti-angiogenic properties of melatonin, serotonin, 3-indolacetic acid, 5-hydroxytryptophol and hydroxytyrosol was mediated by the inhibition of PLCγ1, the main protein involved in the cell proliferation. The results showed that after VEGF stimulation, PLCγ1 became phosphorylated, but pre-incubating the cells with the indolic compounds caused no significant differences in the pPLCγ1/PLCγ1 ratio compared to the positive control with only VEGF (Figure 3A–C). For this reason, the present data support that these compounds’ inhibitory effects on angiogenesis are not mediated by PLCγ1 inhibition. Interestingly, hydroxytyrosol proved to inhibit PLCγ1 phosphorylation (41% inhibition) (Figure 3D,E) which, until then, had not been referenced.

**Figure 4:**
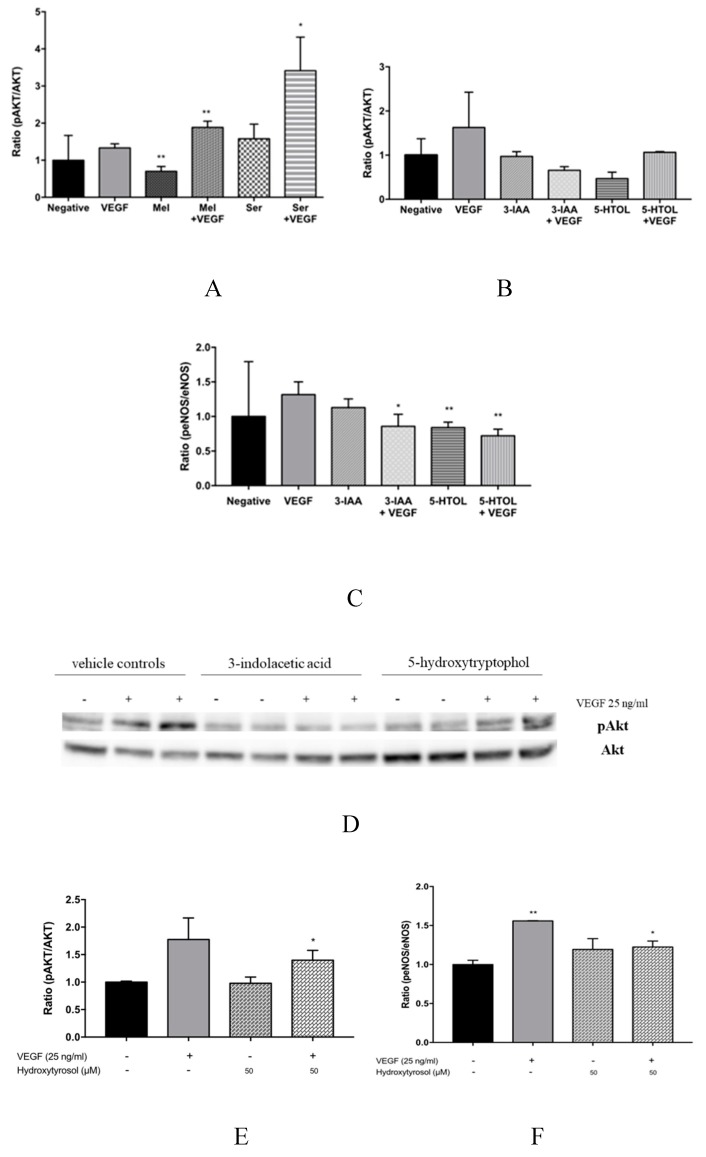
Melatonin and serotonin treatment significantly enhanced VEGF-induced Akt phosphorylation; hydroxytyrosol significantly increased pAkt/Akt and peNOS/eNOS ratios. HUVECs were treated with (**A**) melatonin (1 mM) and serotonin (1 mM); (**B**–**D**) 3-indolacetic acid (1 mM) and 5-hydroxytryptophol (1 mM); and (E and **F**) hydroxytyrosol (50 μM) for 4 h and then incubated with VEGF (25 ng/mL) for 60 min. Western blot membranes were incubated with anti-Akt and anti-p-Akt antibodies and anti-eNOS and anti-p-eNOS antibodies. Data representations of the p-Akt/Akt and peNOS/eNOS ratios are expressed as the mean ± SD (*n* = 4). * *p* <0.05; ** *p* < 0.01 versus negative control. IAA: 3-indolacetic acid; 5-HTOL: 5-hydroxytryptophol; Mel: melatonin; Ser: serotonin.

Secondly, the effect of these compounds on Akt and eNOS activation, which are proteins activated later in the VEGF signalling cascade, was evaluated. In particular, eNOS modulates the nitric oxide generation (NO) via Akt phosphorylation [34,35]. The results obtained in this work demonstrate that VEGF alone activates Akt and eNOS phosphorylation (Figure 4). Additionally, melatonin and serotonin treatment significantly enhanced VEGF-induced Akt phosphorylation and maintained the peNOS/eNOS ratio in the presence of VEGF (Figure 4A, and Table 2). In contrast, 3-indole acetic acid and 5-hydroxytryptophol inhibited VEGF-induced Akt and eNOS phosphorylation (Figure 4B–D). This work’s results also showed that hydroxytyrosol significantly increased both Akt and eNOS phosphorylation (Figure 4E,F).

### 3.4. Effects of Combined Compounds on the Inhibition of VEGF-Induced VEGFR2 Activation

The human diet consists of a great variety of foods, including ones that are sources of these bioactive compounds under study. The cocktail effect of 3-indolacetic acid, melatonin, 5-hydroxytryptophol, serotonin and hydroxytyrosol on the anti-VEGF effect by determining the inhibition of VEGFR-2 phosphorylation was, therefore, evaluated. The results showed that the combination of the indolic compounds did not improve the inhibition percentage of VEGFR-2 phosphorylation (Table 1). However, the combination of melatonin and hydroxytyrosol significantly improved the inhibition percentage of VEGFR-2 phosphorylation compared with both compounds alone (Table 1).

## 4. Discussion

A number of polyphenol compounds usually synthesised in plant foods, such as EGCG, quercetin or dp4 procyanidins, have been shown to directly inhibit the VEGF molecule as an anti-angiogenic molecular mechanism [16]. Hydroxytyrosol has also previously been demonstrated to also inhibit VEGF-induced VEGFR-2 activation, cell proliferation, cell migration and tubular formation in HUVECs [18]. However, both its molecular target and its underlying molecular mechanism are still unknown. In this study, data are presented for the first time supporting that extra- and intra-cellular interactions between hydroxytyrosol and components of the cell surface (VEGFR-2, neurophilins, etc.) could be proposed as a molecular mechanism to explain its anti-angiogenic effect (Figure 1). Therefore, despite hydroxytyrosol being a polyphenol compound, it does not share the same anti-angiogenic molecular mechanism of directly binding to VEGF; rather, it interacts with cell membrane constituents and prevents VEGF from activating its receptor VEGFR-2 in a similar manner to serotonin, 3-indolacetic acid, melatonin and 5-hydroxytryptophol [19].

Lamy et al. [18] determined that pre-stimulating cells with hydroxytyrosol for 24 h inhibited the phosphorylation of VEGFR-2 in Tyr1175 residue with an IC_50_ of 30 μM. Since the half-life of hydroxytyrosol in plasma is 2.43 h [36], the results obtained in this work—pre-stimulating for 4 h only—are more likely to occur in vivo. Thus, IC_50_ was calculated under the 4 h pre-stimulation conditions and resulted at a concentration of 72.4 μM. When compared to the indolic compounds under study (Table 1) which share a similar molecular mechanism, hydroxytyrosol exhibits the highest anti-angiogenic potential, achieving similar pVEGFR-2 inhibition values at concentrations 1000 times smaller.

Interestingly, endothelial cell migration is essential for the initial state of angiogenesis [37]. Therefore, this paper describes for the first time that both serotonin and 5-hydroxytryptophol are potent anti-angiogenic molecules (97% and 50% of migration inhibition, respectively, at 1 mM; Figure 2). This research group has previously demonstrated that melatonin and 3-indolacetic acid, under the same conditions (4 h pre-incubation treatment and at 1 mM), also possess a potent anti-migratory effect on HUVECs [19]. Therefore, the indolic anti-migratory effect, based on their cell migration inhibition percentages, could be ranked as follows: 3-indolacetic acid (99%) [19] > serotonin (97%) (Figure 2) > melatonin (87%) [19] > 5-hydroxytryptophol (50%) (Figure 2). Considering the inhibition percentage of VEGFR-2 phosphorylation of the different indolic compounds under study (3-indolacetic acid (54%) > serotonin (32%) > melatonin (30%) > 5-hydroxytryptophol (23%)) [19] and the ranking of their anti-migratory effect, it was observed that they present an identical compound order. However, the migration values were observed to be higher than the inhibition percentage of pVEGFR-2 in all cases, suggesting that the inhibition of VEGFR-2 phosphorylation is not the only mechanism underlying their anti-angiogenic activity. Indeed, other authors have demonstrated that melatonin inhibits angiogenesis by modifying the expression of intracellular proteins, such as p53 and Bax/Bcl-2, blocking the cellular cycle, inducing apoptosis [38] and reducing cellular proliferation through intracellular proteins (ERK1/2, PI3k, Akt, PKC, NF-kB) mediated by melatonin receptors MT1 and MT2 [39]. Similarly, 3-indolacetic acid, serotonin and 5-hydroxytryptophol could also be modulating these or similar proteins while inhibiting cell migration. Moreover, the effect of hydroxytyrosol on HUVEC migration has been previously described, showing a 59% inhibition rate, although the concentration tested (50 µM) was lower.

Vascular endothelial growth factor-induced VEGFR-2 activation initiates a complex intracellular regulation cascade involving a large number of endogen molecule angiogenic signalling transduction. The biological role of PLCγ1, the first component of the principal VEGFR-2 pathway, has been described in modulating both migration and cell proliferation [40,41,42]. For this reason, this work evaluated whether the inhibition of cell migration in the presence of 3-indolacetic acid, melatonin, 5-hydroxytryptophol, serotonin and hydroxytyrosol was somehow mediated by PLCγ1 inhibition, as has been demonstrated for other bioactive compounds such as epigallocatechin gallate and procyanidin tetramers [16]. The present data (Figure 3A) suggest that all of the compounds tested except hydroxytyrosol failed to deactivate PLCγ1. The results, therefore, demonstrate for the first time that hydroxytyrosol’s anti-angiogenic effect is mediated by the inhibition of PLCγ1 phosphorylation. In fact, its VEGFR-2 phosphorylation inhibition percentage (36.69% of inhibition) (Table 1) and PLC γ1 (41% of inhibition) (Figure 3B) are quite similar. For the indolic compounds, three pathways might be involved: Shb-Sck-PI9K-Rac-IQGAPI, p38/MAPK-HSP27 and TSAd/Src, all of which are also related to cell migration [33]. Since all of the compounds studied have demonstrated their ability to inhibit VEGFR-2 phosphorylation on the Tyr1175 residue [19], the most probable pathway would be Shb-Sck-PI9K-Rac-IQGAPI, mediated by the abovementioned tyrosine residue. Nevertheless, future works would need to be conducted in order to confirm the correct molecular pathway.

Vasodilation is also a VEGF-regulated function through VEGFR-2 activation. This activates eNOS, via Akt, stimulating the formation of NO [34,35]. Certain anti-VEGF drugs, such as bevacizumab, sorafenib and sunitinib, currently employed in cancer therapies, have been shown to increase patients’ risk of suffering hypertension, as a consequence of their VEGF inhibiting effect [14,15,43,44]. However, a number of bioactive compounds such as EGCG and procyanidin tetramers have been demonstrated to not only inhibit VEGF, but also concurrently activate eNOS via Akt [16]. This present study has proved that melatonin and serotonin are able to maintain eNOS phosphorylation (Table 2); hydroxytyrosol significantly activating eNOS (Figure 4F), all three via Akt activation. It may be expected, therefore, that these compounds induce NO bioavailability avoiding the adverse hypertensive effects associated with VEGF inhibitors. To reinforce the results presented here, further research would be necessary in order to evaluate the in vivo effect of these compounds on NO bioavailability.

In the case of VEGF-induced VEGFR-2 phosphorylation, this is the first time that the combination of melatonin and hydroxytyrosol has proved to be more effective in inhibiting VEGF-induced VEGFR-2 activation than using them separately (Table 1). Since melatonin and hydroxytyrosol, when used separately, were also able to maintain and increase, respectively, eNOS phosphorylation, other studies should be performed to evaluate the effect of their combination on eNOS activation.

## Figures and Tables

**Table 1:** Vascular endothelial growth factor (VEGF)-induced VEGFR-2 inhibition percentage (%) of individual and combined compounds.

Compounds	Concentration (mM)	VEGFR-2 Inhibition % (Individual Compounds)	Reference	VEGFR-2 Inhibition % (Combined Compounds)			
				3-Indole Acetic Acid	Serotonin	Melatonin	5-Hydroxytryptophol
3-Indole acetic acid	1	53.56 ± 1.39 IC_50_ = 970.4 µM	[19]	—	36.42 ± 10.31	38.86 ± 6.90	8.89 ± 9.46
Serotonin	1	29.56 ± 14.36			—	43.84 ± 9.21	8.89 ± 9.46
Melatonin	1	32.15 ± 1.87				—	NI
5- Hydroxytryptophol	1	22.99 ± 7.02					—
Hydroxytyrosol	0.05	36.69 ± 6.13 IC_50_ = 72.40 µM (60.32–86.90)	Figure 1B	59.10 ± 8.26	31.03 ± 14.26	48.89 ± 5.61 *	28.26 ± 0.0

Inhibition percentages of VEGFR-2 activation are expressed as the mean ± SD (*n* = 4). * *p* < 0.05 versus individual compounds. The 95% confident intervals of the IC_50_ values are shown in parenthesis. NI: non-inhibition.

**Table 2:** Ratio peNOS/ endothelial nitric oxide synthase (eNOS) values for melatonin and serotonin.

	Ratio peNOS/eNOS Values
Negative	1.35 ± 1.47
VEGF	1.23 ± 0.78
Melatonin	1.05 ± 0.72
Melatonin + VEGF	1.51 ± 0.92
Serotonin	1.57 ± 0.76
Serotonin + VEGF	2.25 ± 1.06

Experimental conditions are described in Figure 4.

## References

[1] World Health Organization (WHO) (2017). Cardiovascular Disease. http://www.who.int/cardiovascular_diseases/world-heart-day-2017/en/.

[2] World Health Organization (WHO) (2018). Cancer. http://www.who.int/news-room/fact-sheets/detail/cancer.

[3] Celletti F.L., Waugh J.M., Amabile P.G., Brendolan A., Hilfiker P.R., Dake M.D. (2001). Vascular endothelial growth factor enhances atherosclerotic plaque progression. Nat. Med..

[4] Bergers G., Benjamin L.E. (2003). Tumorigenesis and the angiogenic switch. Nat. Rev. Cancer.

[5] Giles F.J. (2001). The vascular endothelial growth factor (VEGF) signaling pathway: A therapeutic target in patients with hematologic malignancies. Oncologist.

[6] Dulak J. (2005). Nutraceuticals as anti-angiogenic agents: Hopes and reality. J. Physiol. Pharmacol. Suppl..

[7] Cebe-Suarez S., Zehnder-Fjallman A., Ballmer-Hofer K. (2006). The role of VEGF receptors in angiogenesis; complex partnerships. Cell Mol. Life Sci..

[8] Cook K.M., Figg W.D. (2010). Angiogenesis inhibitors: Current strategies and future prospects. CA-Cancer J. Clin..

[9] Khurana R., Simons M., Martin J.F., Zachary I.C. (2005). Role of angiogenesis in cardiovascular disease—A critical appraisal. Circulation.

[10] Senger D.R., Van De Water L., Brown L.F., Nagy J.A., Yeo K.-T., Yeo T.-K., Berse B., Jackman R.W., Dvorak A.M., Dvorak H.F. (1993). Vascular permeability factor (VPF, VEGF) in tumor biology. Cancer Metast. Rev..

[11] Ferrara N., Kerbel R.S. (2005). Angiogenesis as a therapeutic target. Nature.

[12] Roviello G., Bachelot T., Hudis C.A., Curigliano G., Reynolds A.R., Petrioli R., Generali D. (2017). The role of bevacizumab in solid tumours: A literature based meta-analysis of randomised trials. Eur. J. Cancer..

[13] Varella L., Abraham J., Kruse M. (2017). Revisiting the Role of Bevacizumab in the Treatment of Breast Cancer. Semin. Oncol..

[14] Li M., Kroetz D.L. (2018). Bevacizumab-induced hypertension: Clinical presentation and molecular understanding. Pharmacol. Ther..

[15] Zhu X., Wu S., Dahut W.L., Parikh C.R. (2007). Risks of proteinuria and hypertension with Bevacizumab, an antibody against vascular endothelial growth factor: Systematic review and meta-analysis. Am. J. Kidney Dis..

[16] Moyle C.W.A., Cerezo A.B., Winterbone M.S., Hollands W.J., Aleexev Y., Needs P.W., Kroon P.A. (2015). Potent inhibition of VEGFR-2 activation by tight binding of green tea epigallocatechingallate and apple procyanidins to VEGF: Relevance to angiogenesis. Mol. Nutr. Food Res..

[17] Cerezo A.B., Winterbone M.S., Moyle C.W.A., Needs P.W., Kroon P.A. (2015). Molecular structure-function relationship of dietary polyphenols for inhibiting VEGF-induced VEGFR-2 activity. Mol. Nutr. Food Res..

[18] Lamy S., Ouanouki A., Béliveau R., Desrosiers R.R. (2014). Olive oil compounds inhibit vascular endothelial growth factor receptor-2 phosphorylation. Exp. Cell Res..

[19] Cerezo A.B., Hornedo-Ortega R., Álvarez-Fernández M.A., Troncoso A.M., García-Parrilla M.C. (2017). Inhibition of VEGF-induced VEGFR-2 activation and HUVEC migration by melatonin and other bioactive indolic compunds. Nutrients.

[20] Fernandez-Cruz E., Cerezo A.B., Cantos-Villar E., Richard T., Troncoso A.M., Garcia-Parrilla M.C. (2019). Inhibition of VEGFR-2 Phosphorylation and Effects on Downstream Signaling pathways in Cultivated Human Endothelial Cells by Stilbenes from *Vitis* spp.. J. Agric. Food Chem..

[21] Piñeiro Z., Cantos-Villar E., Palma M., Puertas B. (2011). Direct liquid chromatography method for the simultaneous quantification of hydroxytyrosol and tyrosol in red wines. J. Agric. Food Chem..

[22] Vissers M.N., Zock P.L., Roodenburg A.J.C., Leenen R., Katan M.B. (2002). Olive oil phenols are absorbed in humans. J. Nutr..

[23] Dubbels R., Reiter R.J., Klenke E., Goebel A., Schnakenberg E., Ehlers L., Schiwara H.W., Schloot W. (1995). Melatonin in edible plants identified by radioimmunoassay and by high performance liquid chromatography-mass spectrometry. J. Pineal Res..

[24] Manchester L.C., Tan D.X., Reiter R.J., Park W., Monis K., Qi W. (2000). High levels of melatonin in the seeds of edible plants: Possible function in germ tissue protection. Life Sci..

[25] Maldonado M.D., Moreno H., Calvo J.R. (2009). Melatonin present in beer contributes to increase the levels of melatonin and antioxidant capacity of the human serum. Clin. Nutr..

[26] Hornedo-Ortega R., Cerezo A.B., Troncoso A.M., Garcia-Parrilla M.C., Mas A. (2016). Melatonin and other tryptophan metabolites produced by yeasts: Implications in cardiovascular and neurodegenerative diseases. Front. Microbiol..

[27] De la Puerta C., Carrascosa-Salmoral M.P., García-Luna P.P., Lardone P.J., Herrera J.L., Fernández-Montesinos R., Guerrero J.M., Pozo D. (2007). Melatoninis a phytochemical in olive oil. Food Chem..

[28] Rodriguez-Naranjo M.I., Gil-Izquierdo A., Troncoso A.M., Cantos-Villar E., García-Parrilla M.C. (2011). Melatonin is synthesised by yeast during alcoholic fermentation in wines. Food Chem..

[29] Fernández-Cruz E., Álvarez-Fernández M.A., Valero E., Troncoso A.M., Garcia-Parrilla M.C. (2016). Validation of an analytical method to determine melatonin and compounds related to L-tryptophan metabolism using UHPLC/HRMS. Food Anal. Methods.

[30] Fernández-Cruz E., Álvarez-Fernández M.A., Valero E., Troncoso A.M., Garcia-Parrilla M.C. (2017). Melatonin and derived L-tryptophan metabolites produced during alcoholic fermentation by different wine yeast strains. Food Chem..

[31] Helander A., Wikström T., Löwenmo C., Jacobsson G., Beck O. (1992). Urinary excretion of 5-hydroxyindole-3-acetic acid and 5-hydroxytryptophol after oral loading with serotonin. Life Sci..

[32] Reddy M.Y., Jagota A. (2015). Melatonin has differential effects on age-induced stoichiometric changes in daily chronomics of serotonin metabolism in SCN of male Wistar rats. Biogerontology.

[33] Olsson A.-K., Dimberg A., Kreuger J., Claesson-Welsh L. (2006). VEGF receptor signalling- in control of vascular function. Nat. Rev. Mol. Cell Biol..

[34] Tang F.Y., Nguyen N., Meydani M. (2003). Green tea catechins inhibit VEGF-induced angiogenesis in vitro through suppression of VE-cadherin phosphorylation and inactivation of Akt molecule. Int. J. Cancer.

[35] Chavakis E., Dernbach E., Hermann C., Mondorf U.F., Zeiher A.M., Dimmeler S. (2001). Oxidized LDL inhibits vascular endothelial growth factor induced endothelial cell migration by an inhibitory effect on the Akt/endothelial nitric oxide synthase pathway. Circulation.

[36] Miro-Casas E., Covas M.I., Farre M., Fito M., Ortuño J., Weinbrenner T., Roset P., De La Torre R. (2003). Hydroxytyrosol disposition in human. Clin. Chem..

[37] Alvarez-García V., González A., Alonso-González C., Martínez-Campa C., Cos S. (2013). Antiangiogenic effects of melatonin in endothelial cell cultures. Microvasc. Res..

[38] Cui P., Luo Z., Zhang H., Su Y., Li A., Li H., Zhang J., Yang Z., Xiu R. (2006). Effect and mechanism of melatonin’s action on the proliferation of human umbilical vein endothelial cells. J. Pineal Res..

[39] Cui P., Yu M., Lou Z., Dai M., Han J., Xiu R., Yang Z. (2008). Intracellular signaling pathways involved in cell growth inhibition of human umbilical vein endothelial cells by melatonin. J. Pineal Res..

[40] Takahashi T., Yamaguchi S., Chida K., Shibuya M. (2001). A single autophosphorylation site on KDR/Flk-1 is essential for VEGF-A-dependent activation of PLCγ-1 and DNA synthesis in vascular endothelial cells. EMBO J..

[41] Wu L., Mayo K.D., Dunbar J.D., Kessler K.M., Baerwald M.R., Jaffe E.A., Wang D., Warren R.S., Donner D.B. (2000). Utilization of distinct signaling pathways by receptors for vascular endothelial cell growth factor and other mitogens in the induction of endothelial cell proliferation. J. Biol. Chem..

[42] Simons M., Gordon E., Claesson-Welsh L. (2016). Mechanisms and regulation of endothelial VEGF receptor signalling. Nat. Rev. Mol. Cell Biol..

[43] Touyz R.M., Herrmann S., Herrmann J. (2018). Vascular toxicities with VEGF inhibitor therapies focus on hypertension and arterial thrombotic events. J. Am. Soc. Hypertens..

[44] Wu S., Chen J.J., Kudelka A., Lu J., Zhu X. (2008). Incidence and risk of hypertension with sorafenib in patients with cancer: A systematic review and meta-analysis. Lancet Oncol..

